# Long-term responders to nivolumab in previously treated advanced renal cell carcinoma: a sub-analysis of meet-URO15 study

**DOI:** 10.1007/s00262-024-03741-2

**Published:** 2024-07-02

**Authors:** Carlo Messina, Martina Catalano, Giandomenico Roviello, Annalice Gandini, Marco Maruzzo, Ugo De Giorgi, Paolo Pedrazzoli, Andrea Sbrana, Paolo Andrea Zucal, Cristina Masini, Emanuele Naglieri, Giuseppe Procopio, Michele Milella, Fabio Catalano, Lucia Fratino, Stefania Pipitone, Riccardo Ricotta, Stefano Panni, Veronica Mollica, Mariella Soraru, Veronica Prati, Francesco Atzori, Marilena Di Napoli, Marco Messina, Franco Morelli, Giuseppe Prati, Franco Nole, Andrea Malgeri, Marianna Tudini, Francesca Vignani, Alessia Cavo, Alessio Signori, Giuseppe Luigi Banna, Pasquale Rescigno, Sebastiano Buti, Sara Elena Rebuzzi, Giuseppe Fornarini

**Affiliations:** 1 Oncology Unit, A.R.N.A.S. Civico Palermo, Palermo, Italy; 2 Department of Health Sciences, Section of Clinical Pharmacology and Oncology, University of Florence, Viale Pieraccini 6, 50139 Florence, Italy; 3 Medical Oncology Unit 1, IRCCS Ospedale Policlinico San Martino of Genova, Genova, Italy; 4 Oncology 1 Unit, Department of Oncology, Istituto Oncologico Veneto IOV - IRCCS, Padova, Italy; 5 Department of Medical Oncology, IRCCS Istituto Romagnolo Per Lo Studio Dei Tumori (IRST) “Dino Amadori”, Meldola, Italy; 6 Dipartimento di Medicina Interna e Terapia Medica, Università Degli Studi di Pavia, Pavia, Italy; 7 Dipartimento Di Oncologia, Fondazione IRCCS Policlinico San Matteo, Pavia, Italy; 8 Oncology Department, Azienda Ospedaliero-Universitaria Pisana, Pisa, Italy; 9 Department of Biomedical Sciences, Humanitas University, Milano, Pieve Emanuele Italy; 10 Department of Oncology, IRCCS Humanitas Research Hospital, Via Manzoni 56, 20089 Milan, Rozzano Italy; 11 Medical Oncology Unit, AUSL-IRCCS of Reggio Emilia, Reggio Emilia, Italy; 12 Division of Medical Oncology, IRCCS Istituto Tumori “Giovanni Paolo II”, Bari, Italy; 13 SS Oncologia Medica Genitourinaria, Fondazione IRCCS Istituto Nazionale Dei Tumori, Milano, Italy; 14 Section of Innovation Biomedicine - Oncology Area, Department of Engineering for Innovation Medicine (DIMI), University of Verona and Verona University and Hospital Trust (AOUI Verona), Verona, Italy; 15 Medical Oncology, Centro di Riferimento Oncologico di Aviano, National Cancer Institute, Aviano, Italy; 16 Azienda Ospedaliero Universitaria di Modena, Modena, Italy; 17 Medical Oncology Unit, Istituto di Ricovero e Cura a Carattere Scientifico (IRCCS) MultiMedica, Milan, Italy; 18 Medical Oncology Unit, ASST - Istituti Ospitalieri Cremona Hospital, Cremona, Italy; 19 Medical Oncology, IRCCS Azienda Ospedaliero-Universitaria di Bologna, Bologna, Italy; 20 U. O. Oncologia, Ospedale Di Camposampiero, Camposampiero, Italy; 21 Medical Oncology Unit, ASL CN 2, Alba-Bra, Italy; 22 Medical Oncology Department, University Hospital, University of Cagliari, Cagliari, Italy; 23 Department of Urology and Gynecology, Istituto Nazionale Tumori IRCCS Fondazione G. Pascale, Naples, Italy; 24 UOC Oncologia Medica, Istituto Fondazione G. Giglio, Cefalù, Italy; 25 Medical Oncology Department, Casa Sollievo Della Sofferenza Hospital, IRCCS, San Giovanni Rotondo, Italy; 26 Department of Oncology, Advanced Technologies AUSL - IRCCS Reggio Emilia, Reggio Emilia, Italy; 27 Medical OncologyDivision of Urogenital & Head & Neck Tumors, IEO, European Institute of Oncology IRCCS, Milano, Italy; 28 Department of Medical Oncology, Fondazione Policlinico Campus Bio-Medico, Roma, Italy; 29 Medical Oncology, St. Salvatore Hospital, L’Aquila, Italy; 30 Division of Medical Oncology, Ordine Mauriziano Hospital, Torino, Italy; 31 Oncology Unit, Villa Scassi Hospital, Genova, Italy; 32 Department of Health Sciences, Section of Biostatistics, University of Genova, Genoa, Italy; 33 Portsmouth Hospitals University NHS Trust, Portsmouth, P06 3LY UK; 34 Faculty of Science and Health, School of Pharmacy and Biomedical Sciences, University of Portsmouth, Portsmouth, P01 2UP UK; 35 Translational and Clinical Research Institute, Centre for Cancer, Newcastle University, Newcastle Upon Tyne, UK; 36 Oncology Unit, University Hospital of Parma, Parma, Italy; 37 Department of Medicine and Surgery, University of Parma, Parma, Italy; 38 Medical Oncology Unit, Ospedale San Paolo, Savona, Italy; 39 Department of Internal Medicine and Medical Specialties (Di.M.I.), University of Genoa, Genoa, Italy

**Keywords:** Metastatic renal cell carcinoma, Nivolumab, Immunotherapy, Long-term response, Prognostic factors

## Abstract

**Background:**

Although nivolumab prolongs overall survival (OS) in pretreated patients with metastatic renal cell carcinoma (mRCC), underlining clinical and biological features of long-term responses are still to be determined. This study aims to investigate clinical and pathological characteristics of mRCC patients who achieved long-term responses during nivolumab treatment.

**Materials and methods:**

A retrospective analysis was performed on mRCC patients receiving nivolumab as second or further therapy line between May 2016 and January 2019 in 34 Italian Oncology Centres. Outcome assessments and logistic regression were performed to evaluate factors influencing long-term responses.

**Results:**

A total of 571 patients with a median age of 61 years (range 17–85) were included in the analysis. With a median follow-up of 22.1 (1.0–89.0) months, 23.1% of patients were 2-year progression-free on treatment with nivolumab, hence they were categorized as long-term responders. Baseline characteristics, including age, gender, and histology, were similar between long- and short-term responders. Karnofsky Performance Status ≥ 80% was significantly associated with long-term response (*p* = 0.02), while bone metastases (*p* = 0.03), International mRCC Database Consortium intermediate-poor risk (*p* < 0.01) and Neutrophil-to-Lymphocyte Ratio ≥ 3.2 (*p* = 0.02) were associate with short-term responses. Long-term responders exhibited a median progression-free survival of 55.0 months *versus* 4.0 months of the short-term responders. The median OS was not reached in long-term responders while it was 17.0 months for short*term responders.

**Conclusion:**

This retrospective analysis sheds light on factors associated with long-term response to nivolumab in mRCC. Understanding these clinical features will be essential for selecting patients who may mostly benefit from immunotherapy.

**Supplementary Information:**

The online version contains supplementary material available at 10.1007/s00262-024-03741-2.

## Introduction

In the last decades, the introduction of immune checkpoint inhibitors (ICIs) alone or in combination with vascular endothelial growth factor receptor tyrosine kinase inhibitor (VEGFR-TKI) provided a paradigm shift in the therapeutic landscape of metastatic renal cell carcinoma (mRCC) [1–6]. Due to the survival benefit over everolimus observed in the randomized phase III Checkmate 025 trial [7], nivolumab was the first in class ICI approved in 2005 for patients with mRCC previously treated with at least a prior VEGFR-TKI. However, despite the survival advantage achieved with this new therapeutic strategy, mRCC is still a lethal disease accounting for a median overall survival (OS) of 25 months. mRCC is a heterogenous disease reflecting different clinical behaviors spanning from an indolent to a rapidly progressive disease. Similarly, the benefit achieved from nivolumab may vary widely from long-term disease control rate to hyper-progression [8–10]. Clinical and biological features underlining long-term response to nivolumab in mRCC are still under investigation [11, 12].

In this analysis of the multicentre retrospective Meet-URO 15 study, we attempt to evaluate the association between clinical characteristics and outcome in long-term to nivolumab among patients with mRCC previously treated with at least a prior VEGFR-TKI.

## Materials and methods

### Patients and treatments

This retrospective study involved patients with previously treated mRCC, who received at least one cycle of nivolumab between May 2016 and January 2019 across 34 Oncology Centers in Italy. Patients should be at least 18 years old, have a histologically confirmed diagnosis of mRCC and have received at least one completed infusion of nivolumab as a second or further treatment line, as standard clinical practice. Patients’ demographics and clinical characteristics were reported. Nivolumab was initially delivered intravenously at a dose of 3 mg/kg every 2 weeks, and then at a fixed dosage of 240 mg every 2 weeks or 480 mg every 4 weeks, in line with local clinical protocols. The treatment was continued until either disease progression or intolerable toxicity. Written informed consent was obtained from each patient. Ethical sanction for this study was secured from the Ethics Regional Ethical Committee of Liguria, under registration number 068/2019, and the research adhered to the principles of the Declaration of Helsinki.

### Outcome assessment and statistical analysis

Tumor assessments were performed every 2–4 months of treatment, according to local clinical practice, or whenever progression was clinically suspected according to Response Evaluation Criteria in Solid Tumors (RECIST) version 1.1 [13]. Progression-free survival (PFS) was defined as the time from treatment initiation to disease progression or death whichever occurred first, while OS was defined as the duration from the beginning of nivolumab to death from any cause or to the final follow-up visit date. In this study, patients remained progression-free for > 24 months while receiving nivolumab were categorized as long-term responders.

Kaplan–Meier method was utilized to estimate both PFS and OS throughout the follow-up period. A *χ*^2^ test was applied to compare the distribution of categorical baseline characteristics between long- and short-term responders. Quantitative data were described using median and range, while qualitative data were presented using numbers and percentages. A logistic regression model was employed to assess the influence of each clinical-pathological variable (age, gender, histological type, prior nephrectomy, Karnofsky Performance Status (KPS), International Metastatic RCC Database Consortium (IMDC) score at diagnosis and at start of nivolumab, neutrophil-to-lymphocyte ratio (NLR), sites of metastases, metastatic at diagnosis, line of nivolumab therapy and type of first line therapy) on the long-term response. Significative variables at univariate analysis were included in the multivariate model. Level of statistical significance was set to 0.05. The analyses were conducted using Stata SE version 18.

## Results

### Patients characteristics

This retrospective analysis included a cohort of 571 patients diagnosed with mRCC treated with nivolumab as second or further line of therapy with a median follow-up of 22.1 months.

**Fig. 1:**
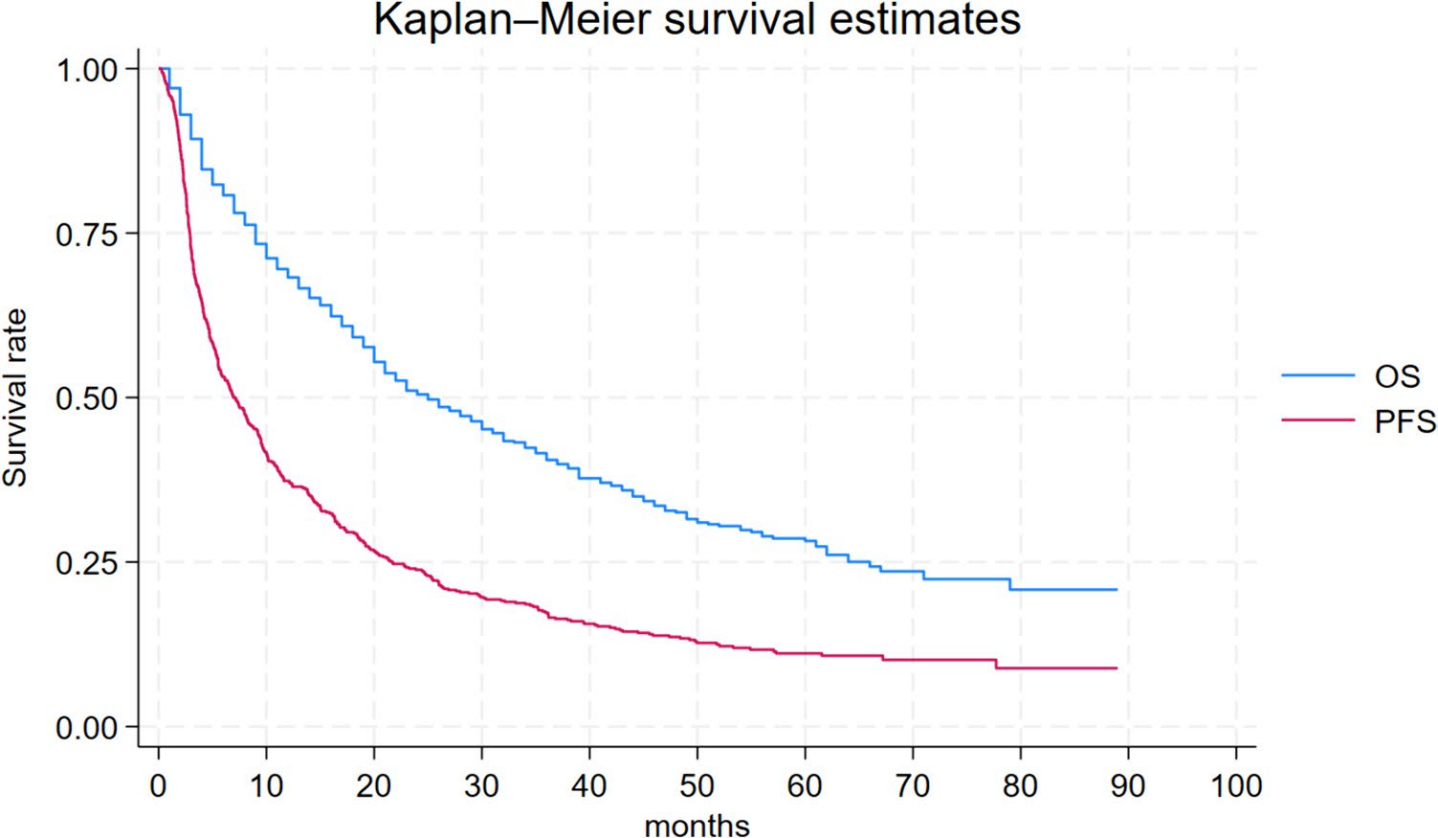
Progression-free survival and overall survival and in entire population

**Fig. 2:**
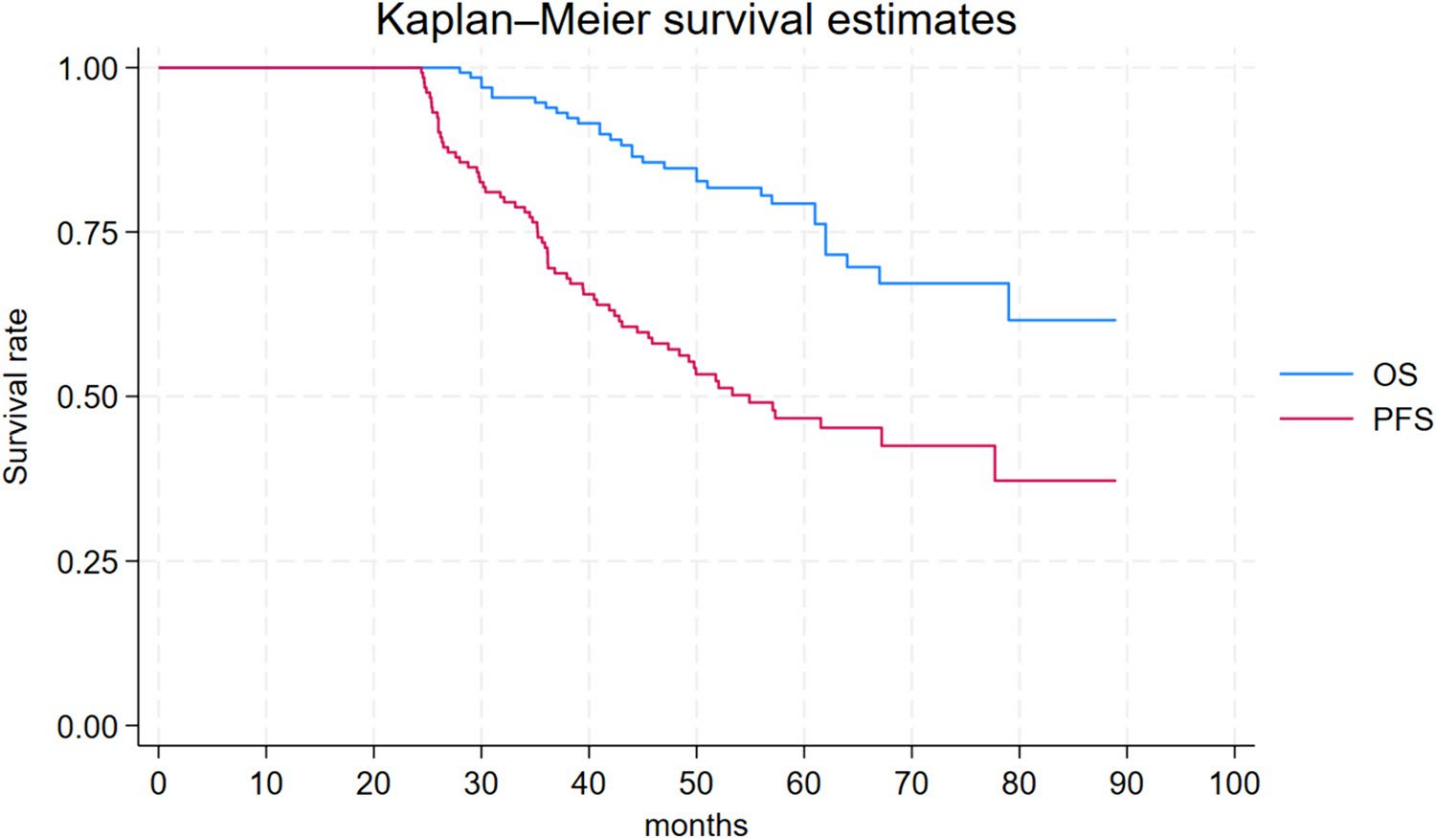
Progression-free survival and overall survival in long-term responders

**Fig. 3:**
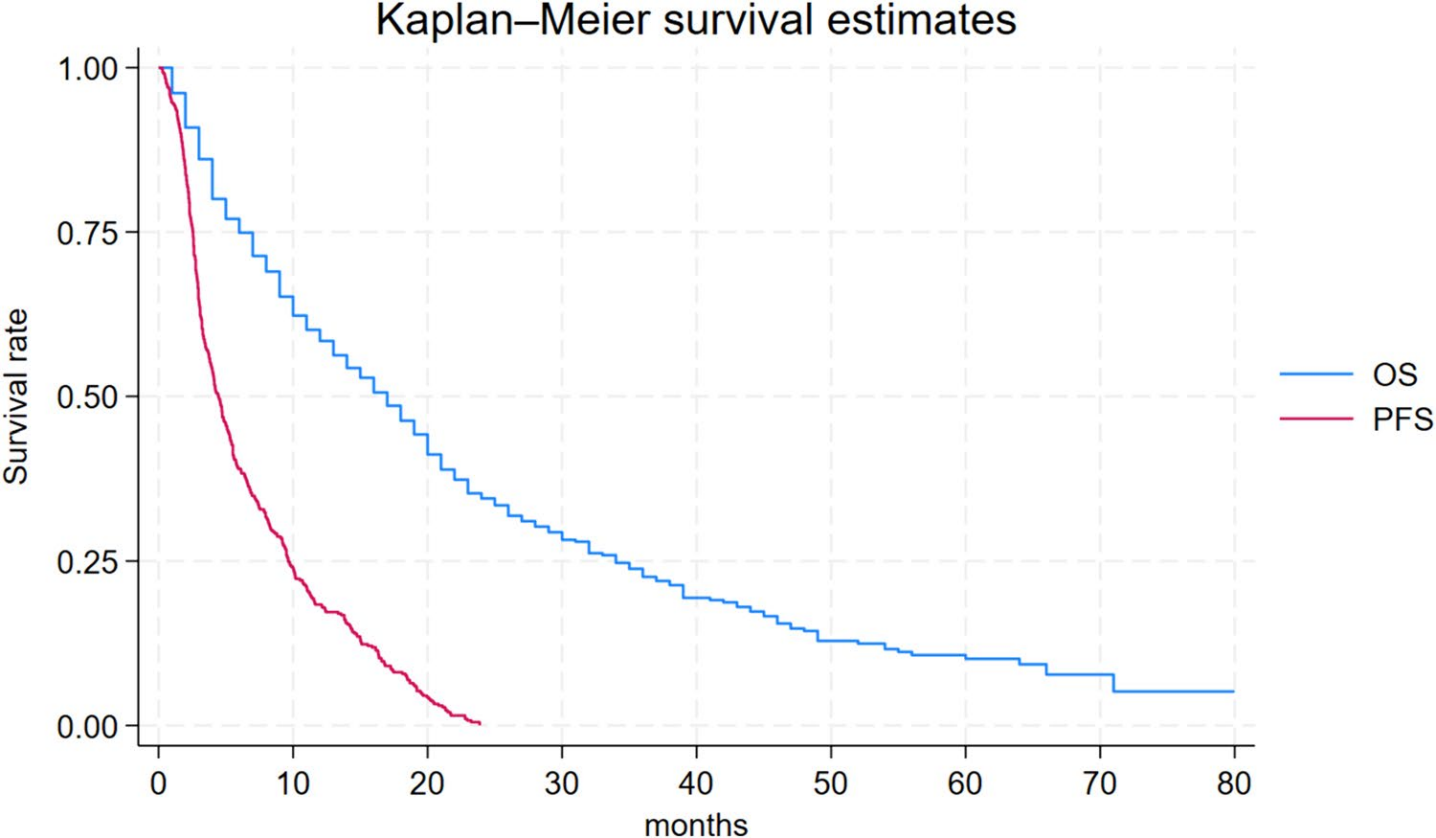
Progression-free survival and overall survival of short-term responders

Among these, 132 patients (23.1%) remained progression-free for > 24 months while receiving nivolumab, and they were categorized as long-term responders. Baseline characteristics according to long-term and short-term responders are detailed in Table 1. Characteristics were well-balanced among the groups in terms of median age, gender distribution, and histology, with clear cell carcinoma being the most prevalent histologic type, accounting for 84.3% of all patients. The median PFS (mPFS) and OS (mOS) for all patients were 7.0 months (95% CI, 5.0–8.0) and 25.0 months (95% CI, 21.0–30.0) respectively (Fig. 1). Long-term responders exhibited a mPFS (mPFS) of 55.0 months (95% CI: 45.0-not reached [NR]), while the median OS was NR (95% CI, 79.0-NR) (Fig. 2). Conversely, short-term responders exhibited a mPFS of 4.4 months (95% CI: 3.9–5.1) with a m OS of 17.0 months (95% CI: 14.0–19.0) (Fig. 3).

**Table 1:** Patients characteristics

	All patients (*N* = 571)	PFS > 24 months (*N* = 132)	PFS ≤ 24 months (*N* = 439)	*P* value
Age, median (range)	61(17–85)	61(32–82)	61(17–85)	0.99 0.99
≥ 70 (%)	151(26.4%)	5(26.5%)	116(26.4%)	
Gender, *n* (%)	402	90	312	0.52
Male	(70.4%)	(68.2)	(71.1%)	
Histology, *n* (%)				
Clear-cell RCC	478(84.3%)	111(84.1%)	367(84.4%)	0.30
Papillary RCC	42(7.4%)	7(5.30%)	35(7.97%)	
Chromophobe RCC	17(2.97%)	7(5.30%)	10(2.27%)	
Sarcomatoid component	30(5.25%)	7(5.30%)	23(5.32%)	
Previous nephrectomy *n* (%)	503	126	377	< 0.01
Yes	(88.1%)	(95.4%)	(85.9)%	
KPS, *n* (%)	478	125	353	< 0.01
≥ 80%	(84.4)%	(94.7)%	(81.3)%	
NLR	234	40	194	< 0.01
≥ 3.2	(41.0)%	(30.3)%	(44.2)%	
IMDC score at diagnosis, *n* (%)	333	66	267	0.02
Intermediate-poor	(66.9)%	(57.4)%	(69.7)%	
Metastatic at diagnosis				
Yes	233	46	187	0.13
	(40.8)%	(34.8)%	(42.6)%	
IMDC score at start of IT, *n* (%)*	427	78	342	< 0.01
Intermediate-poor	(76.8)%	(60.4)%	(81.7)%	
Sites of metastases, *n* (%)				
Lymph-nodal	305 (53.4%)	74 (56.1%)	231 (52.6%)	0.51
Visceral	509 (89.1%)	117 (88.6%)	392 (89.3%)	0.87
Bone	203 (35.5%)	30 (22.7%)	173 (39.4%)	< 0.01
First-line therapy, *n* (%)				
Sunitinib	350 (63.7%)	79 (62.7%)	271 (64.1%)	0.83
Pazopanib	199 (36.2%)	47 (37.3%)	152 (35.9%)	
Nivolumab line, *n* (%)				
Second line	394 (69.0%)	92 (69.7%)	302 (68.8%)	0.91
≥ Third line	177 (31.0%)	40 (30.3%)	137 (31.2%)	

*RCC* renal cell carcinoma, *KPS* Karnofsky performance status, *IMDC* international metastatic RCC database consortium, *IT* immunotherapy.*data available for a total of 556 patients: 129 patients with PFS > 24 months and 427 ≤ 24 months

### Long-term response predictors

Among long-term responders, almost 90% of the patients had previous nephrectomy, with a statistically significant predominance when compared to short-term responders (95% *vs.* 86%; *p* < 0.01). Analysis based on the IMDC risk group displayed a higher percentage of patients with an intermediate-poor risk status at diagnosis in the short-term response group compared to the long-term response group (69.7% *vs.* 57.4%; *p* < 0.01). A NLR ≥ 3.2 was recorded in 30.3% of patients exhibiting a long-term response versus 44.2% of those with short-term responses (*p* < 0.01).

Furthermore, a higher proportion of patients in the short-term responders group presented bone metastases compared to the long-term responders (39.4% *vs*. 22.7%; *p* < 0.01). Notably, no statistically significant differences were noted between the two groups concerning first-line therapy and the number of therapy lines of nivolumab (2nd *vs*. > 3rd line).

Logistic regression analysis was conducted on the entire cohort of 571 patients to explore the associations between clinical and pathological variables and long-term responses. Factors evaluated as potential risks included age, gender, histological type, prior nephrectomy, KPS, IMDC score at diagnosis and at start of nivolumab, NLR, sites of metastases, metastatic at diagnosis, line of nivolumab therapy and type of first line therapy. The odds ratios (OR) estimated for each variable in both univariable and multivariate analyses are presented in Tables 2 and 3.

**Table 2:** Univariate analysis of the relationship of clinical-pathological variables with PFS > 24 months

Variable	Odds ratio	95% CI	*P* value
KPS (≥ 80% vs < 80)	4.10	1.84–9.11	< 0.01
Previous nephrectomy (yes vs no)	3.45	1.46–8.18	0.01
Bone metastasis (yes vs no)	0.45	0.29–0.71	< 0.01
IMDC score at diagnosis (Intermediate-poor vs good)	0.58	0.38–0.90	0.01
IMDC score at start of IT (Intermediate-poor vs good)	0.34	0.22–0.52	< 0.01
NLR(≥ 3.2 vs < 3.2)	0.56	0.37–0.85	< 0.01

*KPS* Karnofsky performance status, *IMDC* International metastatic RCC database consortium, *NLR* Neutrophil–Lymphocyte ratio

**Table 3:** Multivariate analysis of the relationship of various clinical-pathological variables with PFS > 24 months

Variable	Odds ratio	95% CI	*P* value
KPS (≥ 80% vs < 80)	2.68	1.18–6.11	0.02
Bone metastasis (yes vs no)	0.58	0.36–0.94	0.03
IMDC score at start of IT (Intermediate-poor vs good)	0.44	0.28–0.68	< 0.01
NLR (**≥ **3.2 vs < 3.2)	0.59	0.38–0.92	0.02

*KPS* Karnofsky performance status, *IMDC* International metastatic RCC database consortium, *NLR* Neutrophil–Lymphocyte ratio

In the univariate analyses, long-term responders displayed higher odds of having KPS ≥ 80% (OR, 4.10; 95% CI, 1.84–9.11; *p* < 0.01) (Fig. 1S) and having undergone previous nephrectomy (OR, 3.45; 95% CI, 1.46–8.18; *p* = 0.01) (Fig. 2S). Conversely, they exhibited lower odds of having bone metastases (OR, 0.45; 95% CI, 0.29–0.71; *p* < 0.01) (Fig. 3S), an IMDC intermediate-poor status at diagnosis (OR, 0.58; 95% CI, 0.38–0.90; *p* = 0.01) and at the onset of nivolumab treatment (OR, 0.34; 95% CI, 0.22–0.52; *p* < 0.01) (Fig. 4S), as well as an NLR ≥ 3.2 (OR, 0.56; 95% CI, 0.37–0.85; *p* < 0.01) (Fig. 5S) compared to short-term responders.

The multivariable analysis exploring the relationship between clinical-pathological variables and response group is detailed in Table 3. Patients with PFS > 24 months were more likely to have a KPS ≥ 80% (OR, 2.68; 95% CI, 1.18–6.11; *p* = 0.02) and less likely to have bone metastases (OR, 0.58; 95% CI, 0.36–0.94; *p* = 0.03), an IMDC intermediate-poor status at the start of nivolumab (OR, 0.44; 95% CI, 0.28–0.68; *p* < 0.01), and an NLR ≥ 3.2 (OR, 0.59; 95% CI, 0.38–0.92; *p* = 0.02) compared to patients with PFS ≤ 24 months.

## Discussion

As the therapeutic landscape of advanced RCC has changed due to the development and reimbursement of new treatment combinations [1–6], a deeper understanding of clinical characteristics and baseline laboratory features affecting clinical outcomes is needed and may help clinicians in the treatment decision making. Several nomograms for mRCC were developed to better predict prognosis and are mostly based on clinical factors laboratory parameters [14, 15]. These models have many limitations since they have been designed before the approval of ICI and do not take into account prognostic factors such as age, site of metastases, number and duration of previous treatments and inflammatory scores [16].

The phase 3 CheckMate 025 trial showed longer median OS with nivolumab (25 months) compared with everolimus (19.6 months) in previously treated patients with advanced RCC [7]. This benefit was sustained across all the subgroups, including Memorial Sloan Kettering Cancer Center (MSKCC) and IMDC risk groups, number and sites of metastases, age < 65 and ≥ 65 years, number, and duration of prior therapies^15^. The safety and efficacy observed in the CheckMate 025 trial were consistent with those reported in real-world setting major series, showing a good correspondence from the results in clinical trials and those in clinical practice [15, 17]. As such, further investigations of clinical predictive factors that could more accurately define the outcome of advanced RCC in the current treatment landscape from clinical practice remains a clinical need.

The multicentre retrospective Meet-URO 15 study [18] explored the prognostic role of baseline peripheral blood inflammatory indices and clinical factors in advanced RCC patients receiving nivolumab as second or further line to develop a prognostic score that could better predict survival outcome and overcome the limitations of previous analyses in short series with nomograms [14, 15]. Inflammatory indexes as neutrophil–lymphocyte ratio (NLR), lymphocyte-to-monocyte ratio (LMR), platelet-to-lymphocyte ratio (PLR), systemic inflammation index (SII), and systemic inflammation response index (SIRI) have been recently developed and confirmed for outcome prediction in pretreated mRCC [16, 19, 20]. The MeetURO-Score included priori and recent biomarkers showing a prognostic impact for NLR, IMDC score, and bone metastases identifying five different prognostic groups: group 1 (mOS not reached), group 2 (mOS 43.9 months), group 3 (mOS 22.4 months), group 4 (mOS 10.3 months) and group 5 (mOS 3.2 months).

However, although nivolumab provided a survival benefit in pretreated advanced RCC patients [21], usually only a small proportion of them achieves a long-term benefit [8].

In the present analysis of Meet-URO 15 we attempted to define the clinical characteristics that correlate with longer response to nivolumab. At multivariable analysis we found out that patients with PFS > 24 months were more likely to have a KPS ≥ 80% and less likely to have bone metastases, an IMDC intermediate-poor status, and an NLR ≥ 3.2.

Our data are like that reported in a previous long-term response study of sunitinib and pazopanib with accordance to the general characteristics of mRCC patients [22–24].

Age < 65 years, previous nephrectomy, absence of bone or lung metastases and favorable MSKCC risk status were the factors associated with long-term responses in mRCC patients receiving TKI as first line therapy [22–24]. Accordingly with previous studies, NLR is significantly associated with poorer OS and PFS, and lower rates of response and clinical benefit, after ICI therapy across multiple cancer types [25]. We acknowledge several limitations of the study including the retrospective design, and the numbers of previous treatment received. However, we believe that our study provides the rationale for prospectively exploring the presented putative biomarkers of prolonged response to ICI. Given the lack of validated biomarkers, the identification of prognostic and predictive clinical and biochemical features would allow to identify patients that could most benefit from immunotherapy. Moreover, these data are extremely punctual since several ongoing phase III clinical trials are exploring the efficacy of ICI combinations in patients previously progressed to first line ICI-based therapy [26, 27].

## Conclusion

In this large retrospective Meet-URO 15 analysis, we identified, among patients with mRCC suitable for nivolumab treatment, the prognostic role of clinical factors and inflammatory indices that may predict long response to nivolumab in real life setting. Future perspectives include the external validation of these findings in the International multicenter real-world REGistry for patients with metastatic renAL cell carcinoma—Meet-URO 33 study (REGAL study) [28].

### Supplementary Information

Below is the link to the electronic supplementary material.

Supplementary file 1 (DOCX 16538 kb)

## Data Availability

The datasets generated during and/or analyzed during the current study are available from the corresponding author on reasonable request.
