# Supplementary material for: Long-term responders to nivolumab in previously treated advanced renal cell carcinoma: a sub-analysis of meet-URO15 study
Source: Cancer Immunol Immunother. 2024 Jul 2;73(9):161. doi: 10.1007/s00262-024-03741-2 (PMC11219688; doi:10.1007/s00262-024-03741-2)
Supplement: Supplementary file 1 — (DOCX 16538 kb) [file 262_2024_3741_MOESM1_ESM.docx]

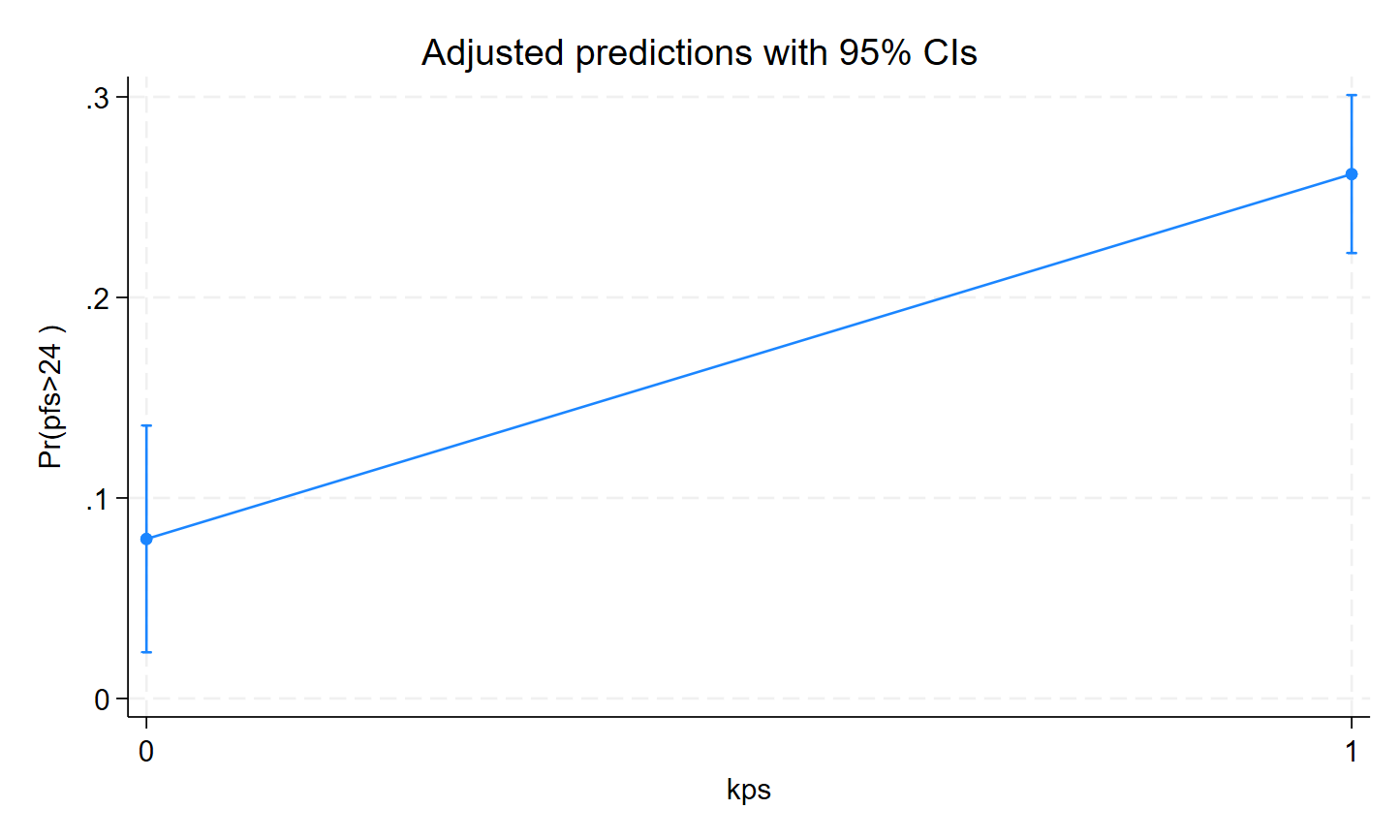


**Figure 1S:** Correlation between KPS and long-term responses. Long responders displayed higher odds of having KPS≥80% (OR 4.10; 95% CI, 1.84-9-11; p<0.01)


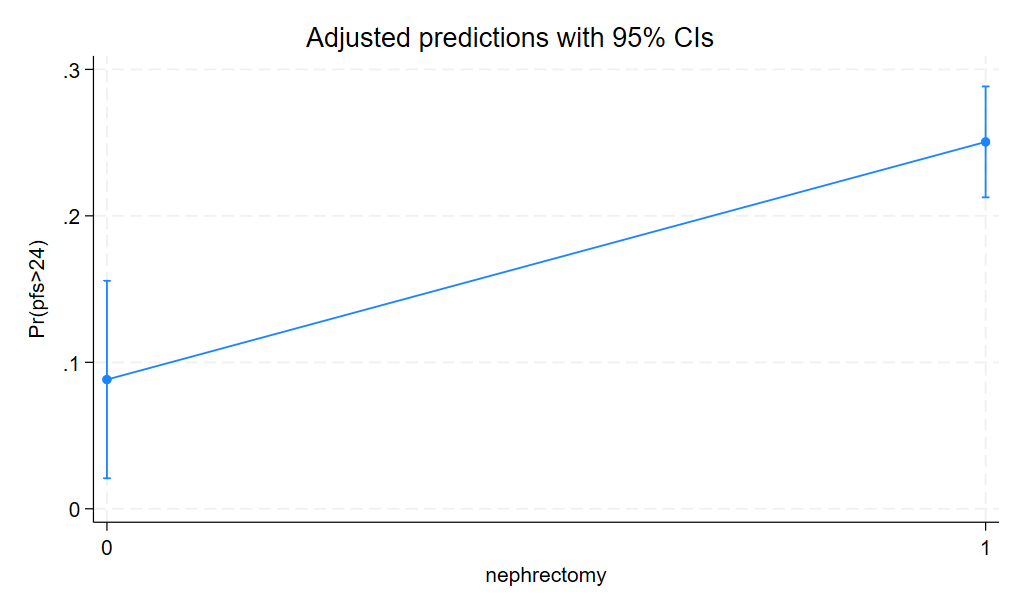


**Figure 2S:** Correlation between previous nephrectomy and long-term response. Long responders displayed higher odds of having undergone previous nephrectomy (OR, 3.45; 95% CI, 1.46–8.18; p = 0.01)


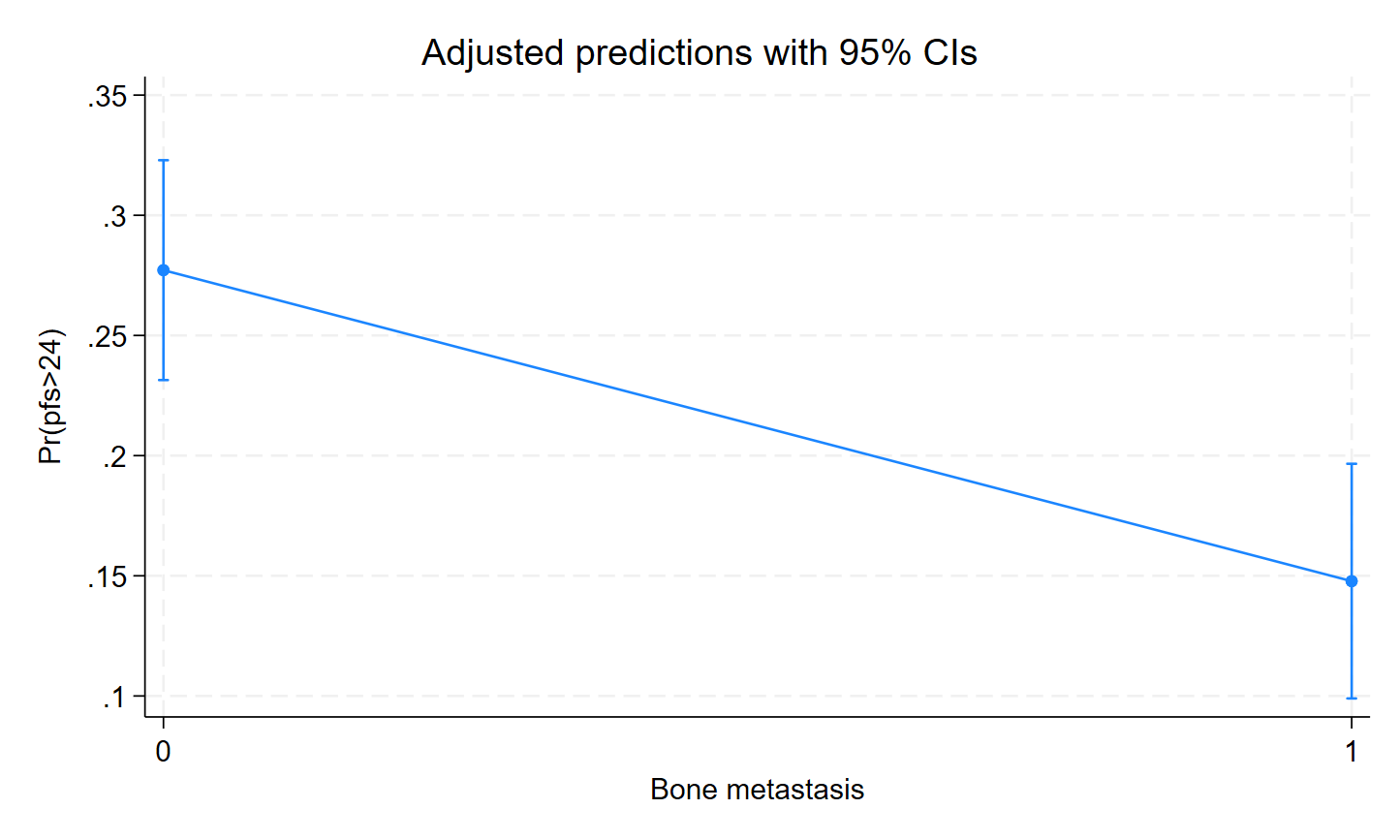


**Figure 3S:** Correlation between bone metastases and long-term response. Long responders displayed lower odds of having bone metastases (OR, 0.45; 95% CI, 0.29–0.71; p < 0.01)


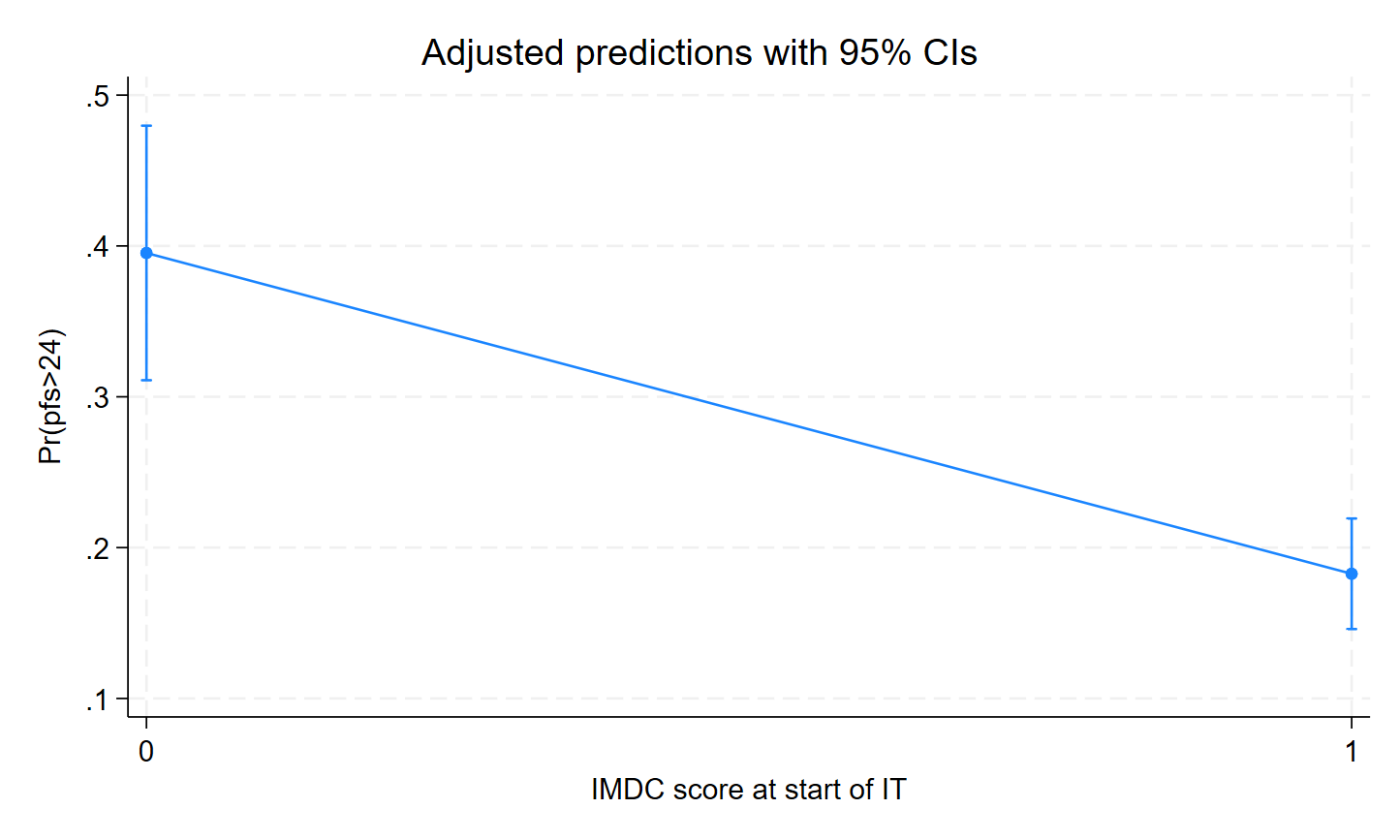


**Figure 4S**: Correlation between long-term response and IMDC. Long responders displayed lower odds of having intermediate-poor IMDC at the onset of nivolumab treatment (OR, 0.34; 95% CI, 0.22-0.52; p<0.01)


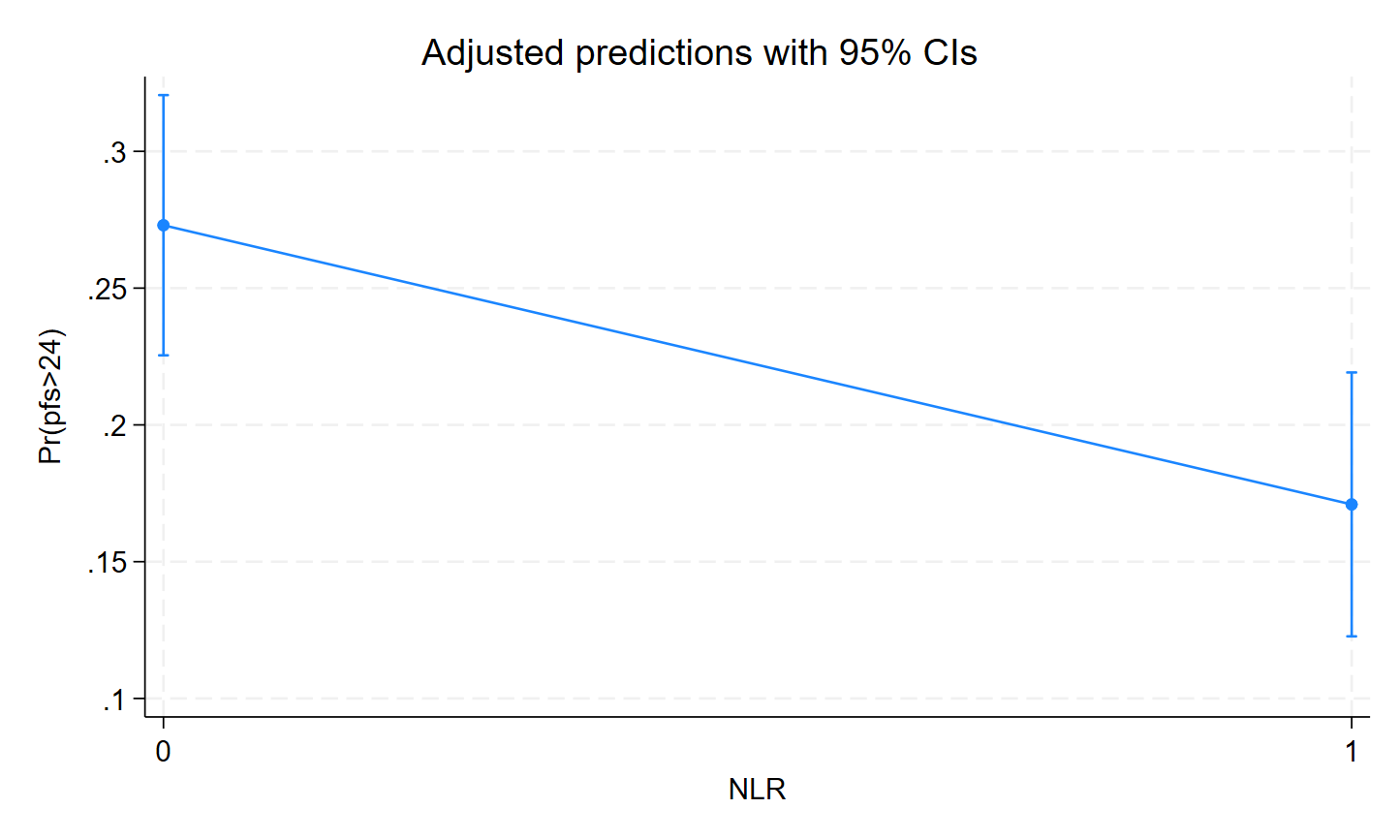


**Figure 5S**: Correlation between long-term response and NLR. Long responders displayed lower odds of having NLR ≥3.2 (OR, 0.56; 95% CI, 0.37-0.85; p<0.01)
